# Total Colonic Decompression Fails to Decrease Post-Colonoscopy Abdominal Pain: A Double-Blind Randomized Controlled Trial

**DOI:** 10.5152/tjg.2026.25559

**Published:** 2026-01-15

**Authors:** Ekrem Aslan, Mahmut Gokhan Teker, Suleyman Bas, Binnur Pinarbasi Simsek

**Affiliations:** 1 Department of Internal Medicine, İstinye University Faculty of Medicine, İstanbul, Türkiye; 2 Department of Gastroenterology, Liv Hospital Ulus, İstanbul, Türkiye; 3 Department of Anesthesiology and Reanimation, İstinye University Faculty of Medicine, İstanbul, Türkiye; 4 Department of Internal Medicine, Sancaktepe Şehit Prof. Dr. İlhan Varank Training and Research Hospital, University of Health Sciences, İstanbul, Türkiye

**Keywords:** Abdominal pain, colonoscopy, total colonic decompression

## Abstract

**Background/Aims:**

Post-colonoscopy pain is among the most common minor side effects of colonoscopy. Total colonic decompression, involving the decompression of intraluminal air from the colon through reinsertion of the colonoscope to the cecum post-colonoscopy, is proposed to reduce this pain. This study aims to evaluate the effect of total colonic decompression on post-colonoscopy abdominal pain.

**Materials and Methods:**

A total of 240 eligible colonoscopy participants were randomized (1 : 1) into the decompression and control groups. In the decompression group, the cecum was reintubated, and the colonoscope was withdrawn with air aspiration post colonoscopy. However, this maneuver was omitted in the control group. Demographics, vital signs, anesthetic doses, procedure duration, and interventions were recorded. Abdominal pain was assessed using a 10-point Visual Analog Scale within 30 minutes and again at 24 hours post-procedure.

**Results:**

The final analysis included 114 participants in the decompression group and 104 in the control group. Clinical and demographic characteristics were comparable between groups. Post-colonoscopy pain occurred in 17 patients (14.9%) in the decompression group and 20 patients (19.2%) in the control group (*P* = .396) within 30 minutes post-procedure. At 24 hours post-procedure, the rates were 2.6% and 3.8%, respectively (*P* = .712). The administered propofol dose (196.5 ± 60.5 vs. 177.3 ± 39.0 mg, *P* = .005) and hypoxia (SpO_2_: 94.9 ± 5.2% vs. 96.4 ± 2.8%, *P* = .006) were significantly higher in the decompression group.

**Conclusion:**

Total colonic decompression does not alleviate post-colonoscopy abdominal pain and is associated with a higher propofol dose, leading to hypoxia.

**Main Points**

- Total colonic decompression does not significantly reduce early or late post-colonoscopy abdominal pain compared with standard colonoscopy.
- Total colonic decompression increases procedural time and requires higher propofol doses, which are associated with an increased risk of hypoxia.
- The presence of comorbid disease appears to have a stronger influence on post-colonoscopy pain than procedural technique.

## Introduction

Colonoscopy is a widely used method for diagnosing and treating various colorectal diseases and remains the gold standard for colon cancer screening.^1^^,^^2^ Although generally safe, major and minor side effects may occur during and after the procedure. Post-colonoscopy abdominal pain (PCAP) is one of the most common minor side effects of colonoscopy and may develop in 48% of colonoscopies, even under conscious sedation.^3^^,^^4^ Concern about PCAP may lead to the avoidance of colonoscopy among patients undergoing colorectal cancer screening.^5^ Various intraprocedural methods, such as the use of carbon dioxide for insufflation, total or segmentary air decompression, and choosing the right lateral position instead of the left lateral decubitus position, as well as postprocedural methods, including using a rectal tube or hot pack therapy, can be employed to prevent the development of PCAP.^6^^-^^10^

The primary cause of PCAP has been reported to be colonic distension caused by insufflated air.^7^ Therefore, aspiration of intraluminal air may prevent the development of PCAP. Total colonic decompression (TCD), defined as the reintroduction of the colonoscope to the cecum after completing a colonoscopy and the aspiration of intraluminal air during withdrawal, is one of the methods used for this purpose. Prior studies in the literature have investigated the impact of TCD on PCAP; however, their findings are conflicting. For example, Lee et al^11^ reported that TCD had no beneficial effect on pain. In contrast, Park et al^12^ reported a significant reduction in PCAP following its application. These discrepancies underscore the need for further investigation. Accordingly, this study aimed to evaluate the impact of TCD on the development of PCAP.

## Materials and Methods

### Study Design and Population

This study was designed as a double-blind, prospective, and randomized controlled trial. Between December 2023 and August 2024, patients referred for colonoscopy from the check-up department were screened for eligibility. The study included patients aged 18 years or older who provided consent to participate. Exclusion criteria included age under 18 years, a history of previous colon surgery, an American Society of Anesthesiology (ASA) risk classification of 3 or higher, diagnosis of colon cancer or inflammatory bowel disease, failure to comply with the bowel preparation regimen, and refusal to provide informed consent. Patients with a cecal intubation time exceeding 10 minutes or who could not be contacted by phone on the post-colonoscopy day were also excluded. The study was approved by the Clinical Research Ethics Committee of the İstinye University (Decision No.: K-72; Date: December 27, 2023). Written informed consent was obtained from all participants in accordance with the principles of the Declaration of Helsinki.

### Colonoscopy Procedure

Patients underwent bowel preparation consisting of a low-fiber diet initiated 48 hours before the procedure and the administration of 2 split-dose sachets of sodium picosulfate with 4000 mL of water on the night before the colonoscopy. All colonoscopies were performed by a single expert gastroenterologist (who had performed 1000 colonoscopies annually) using a single-channel high-definition colonoscope (Olympus CF-HQ190L, Olympus Optical Co. Ltd., Tokyo, Japan), which insufflates room air, with patients positioned in the left lateral decubitus position. When required, the endoscopy technician provided abdominal pressure or assisted in changing the patient’s position at the endoscopist’s request.

Throughout the procedure, patients were continuously monitored for oxygen saturation (SpO_2_), blood pressure, and heart rate. All patients were administered supplemental oxygen at a flow rate of 2-5 L/min. Sedation was administered by an expert anesthesiologist. A standard intravenous bolus of midazolam (2 mg) and remifentanil (25 mcg) was administered, followed 1-2 minutes later by a bolus of propofol (1 mg/kg). Additional doses of propofol (0.5-1 mg/kg) were administered during the procedure as needed, based on the patient’s level of consciousness and pain response. Vital signs and doses of sedative medications administered were recorded throughout the procedure. Reaching the cecum was confirmed by direct contact of the colonoscope tip to the appendiceal orifice, followed by ileal intubation in all patients. In addition, during intubation, polyps measuring less than 10 mm or those considered unlikely to be visualized during withdrawal were removed. The time spent on polypectomy during the intubation phase was included in the time to reach the cecum. In all patients, the colonoscope was withdrawn from the cecum, with the lumen insufflated using room air. In accordance with the European Society of Gastrointestinal Endoscopy (ESGE) guidelines, the withdrawal time was maintained at a minimum of 6 minutes.^13^ Upon reaching the rectum, retroflexion was performed to evaluate the anal canal. At this stage, the sealed envelope previously selected by the patient was opened by the endoscopy technician, and the subsequent procedural step was performed according to the randomization result, as detailed in the Randomization and Blinding section.

The time to reach the cecum and total procedure time (defined as the sum of time to reach the cecum, withdrawal time, and time spent in retroflexion to visualize the anal canal, excluding time spent in the ileum) were recorded for both groups. In the decompression group, the time to re-reach the cecum was also recorded. All biopsies and polypectomies performed during the procedure were documented, including their location and the number of specimens obtained. Any abdominal pressure or position changes during the procedure were also recorded. The quality of bowel preparation was assessed using the Aronchick Scale and classified as excellent, good, fair, poor, or inadequate.^14^

### Randomization and Blinding

Patients who met the inclusion criteria and consented to participate in the study were asked by the recovery-room nurse to select one of the sealed opaque envelopes prepared in equal numbers to match the total number of colonoscopies scheduled for that day. This nurse was responsible for both pre-colonoscopy preparation and post-colonoscopy follow-up. Half of the envelopes were empty, while the other half contained white paper.

After the colonoscopy was completed and retroflexion was performed for evaluation of the anal canal, the sealed envelope brought by the patient was opened by the endoscopy technician in the endoscopy room. If the envelope contained a white paper, the patient was assigned to the decompression group; if it was empty, the patient was assigned to the control group. For patients in the control group, the procedure was concluded at this point. However, for those in the decompression group, after the cecum was reached rapidly without detailed mucosal inspection, luminal air was aspirated, and the colonoscope was withdrawn.

### Pain Assessment

A 10-point VAS was used to evaluate pain following colonoscopy, with a score of zero indicating no pain and a score of 10 representing severe pain. Pain scores were recorded twice: within the first 30 minutes after the patient regained consciousness in the recovery room and 24 hours post-colonoscopy through a follow-up phone call. Pain intensity was categorized as follows: no pain (VAS = 0), mild pain (VAS = 1-3), moderate pain (VAS = 4-6), and severe pain (VAS ≥ 7).^15^

### Statistical Analysis

To evaluate the statistical power of the study, a power analysis was conducted using the G*Power 3.1.9.7 software (Heinrich Heine University; Düsseldorf, Germany), employing the Wilcoxon–Mann–Whitney test for 2 independent groups. The power analysis revealed that a minimum of 92 participants per group, yielding a total recommended sample size of 184 participants, was required to achieve a statistical power of 95%. This indicates a 95% probability of detecting a true difference in the prevalence of abdominal pain, if one exists. Furthermore, the effect size (Cohen’s *d* = 0.5) represented a medium effect, suggesting that differences of this magnitude are likely to be detected.

Statistical analyses were performed using IBM SPSS Statistics version 25.0 (IBM SPSS Corp.; Armonk, NY, USA)). Normality was assessed with Kolmogorov–Smirnov and Shapiro–Wilk tests. Normally distributed variables were expressed as mean ± SD; non-normal variables as median (interquartile range). Group comparisons were made using the Independent Samples *t*-test for parametric data and the Mann–Whitney *U*-test for non-parametric data. Categorical variables were analyzed using the Chi-square or Fisher’s Exact test when appropriate. Univariate and multivariate logistic regression analyses were conducted to identify factors associated with PCAP, with results reported as odds ratios (ORs), 95% CIs, and *P*-values.

## Results

**Figure 1.**
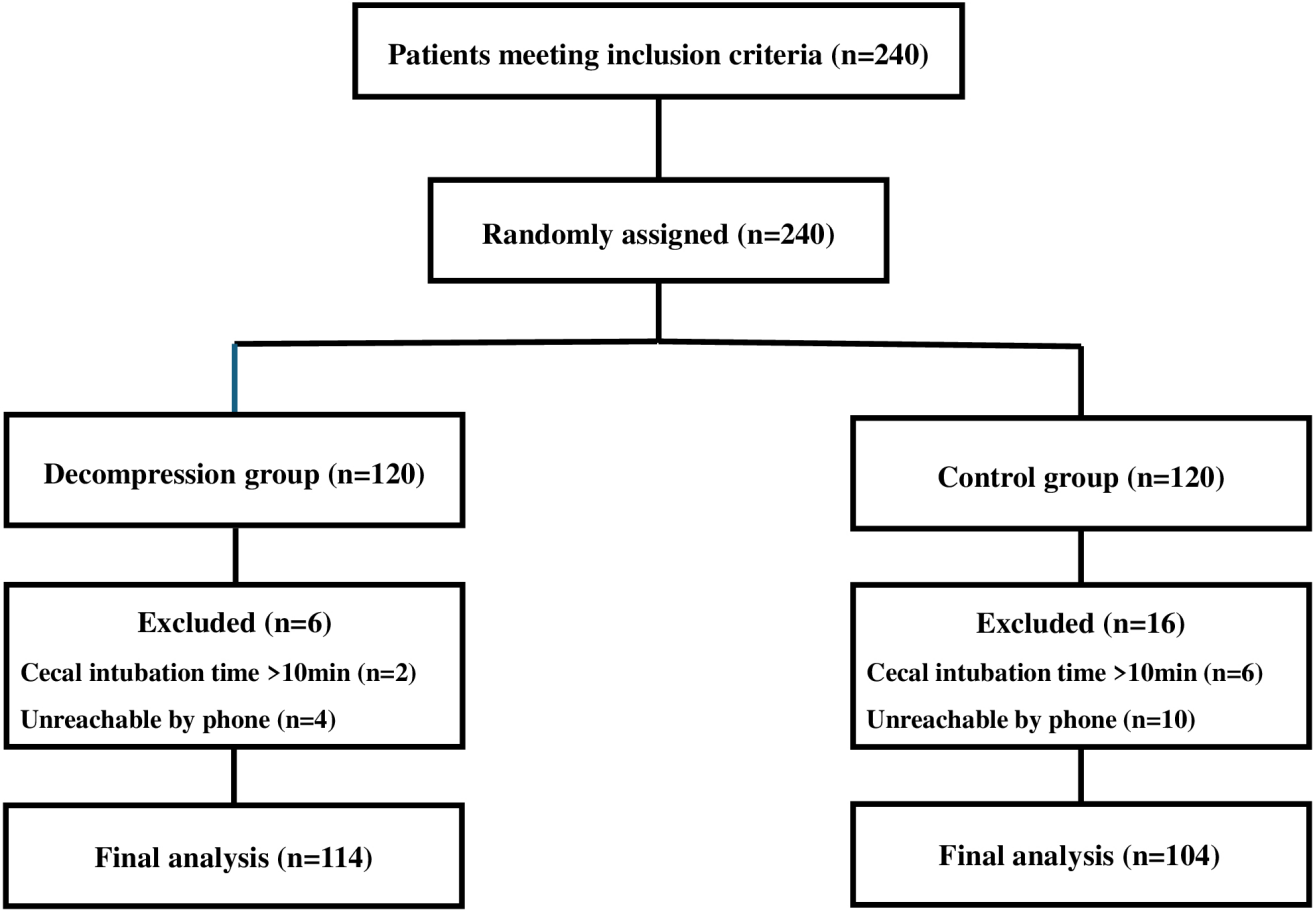
Flowchart of participants and study design.

A total of 240 patients who met the inclusion criteria were randomly assigned to the decompression and control groups. Six patients were excluded from the decompression group, and 16 were excluded from the control group. The final analysis was conducted with 114 participants in the decompression group and 104 in the control group (Figure 1).

### Patient Demographics

The demographic, clinical, and colonoscopic characteristics of the 2 research groups are presented in Table 1. There were no statistically significant differences between the decompression and control groups regarding age, sex, body mass index (BMI), smoking status, comorbidities, history of previous abdominal or gynecological surgery, or presence of irritable bowel syndrome (IBS) (*P* > .05).

### Anesthesia-Related Parameters

The dose of propofol administered for sedation was significantly higher in the decompression group (196.54 ± 60.50 mg vs. 177.26 ± 38.98 mg,* P* = .005). The mean lowest SpO_2_ value in the decompression group was significantly lower than in the control group (94.9 ± 5.2% vs. 96.4 ± 2.8%, *P* = .006). SpO_2_ levels decreased to 80%-90% in 3 patients in the control group and in 8 patients in the decompression group. In comparison, a transient desaturation below 80% was observed in 1 patient in the decompression group. None of these events resulted in adverse outcomes or necessitated clinical action. No statistically significant differences were observed between the groups in terms of other vital signs (heart rate, blood pressure) and the highest SpO_2 _value (*P* > .05).

### Procedure-Related Parameters

There was no statistically significant difference between the decompression and control groups in terms of bowel preparation (fair, good, or excellent: 21.1%, 35.1%, 45.9% vs. 20.2%, 42.3%, 37.5%, respectively; *P* = .524). There were no significant differences between the 2 groups regarding total procedure time (737 [580.75-891.75] vs. 738 [612.25-931.75] seconds, *P *= .327) or cecum reaching time (203 [141-277.50] vs. 222.50 [153-318.25] seconds, *P* = .089). The median reinsertion time in the decompression group was 109.50 [80.75-167] seconds. No statistically significant differences were observed between the decompression and control groups in the rates of colonic biopsy (11.4% vs. 7.7%, *P* = .354) and polypectomy (33.3% vs. 38.2%, *P* = .685). The need for abdominal compression was significantly lower in the decompression group (8.8% vs. 19.2%, *P* = .025). None of the patients required a positional change to achieve cecal intubation.

### Post-Colonoscopy Abdominal Pain

The overall incidence of PCAP following colonoscopy was 16.9% (37/218) among all enrolled patients. Early PCAP (within 30 minutes after colonoscopy) was reported in 14.9% of patients in the decompression group, and 19.2% in the control group (*P* > .05). Pain severity was similar between groups, with mean VAS scores of 0.52 ± 1.42 in the decompression group and 0.93 ± 2.16 in the control group (*P* > .05).

In terms of pain severity, 10 patients (58.8%) in the decompression group experienced mild, 5 (29.4%) had moderate, and 2 (11.8%) had severe pain. In the control group, the values were 6 (30%) for mild, 9 (45%) for moderate, and 5 (25%) for severe pain. As presented in Table 2, there was no statistically significant difference between the groups in terms of pain severity (*P* > .05).

**Figure 2.**
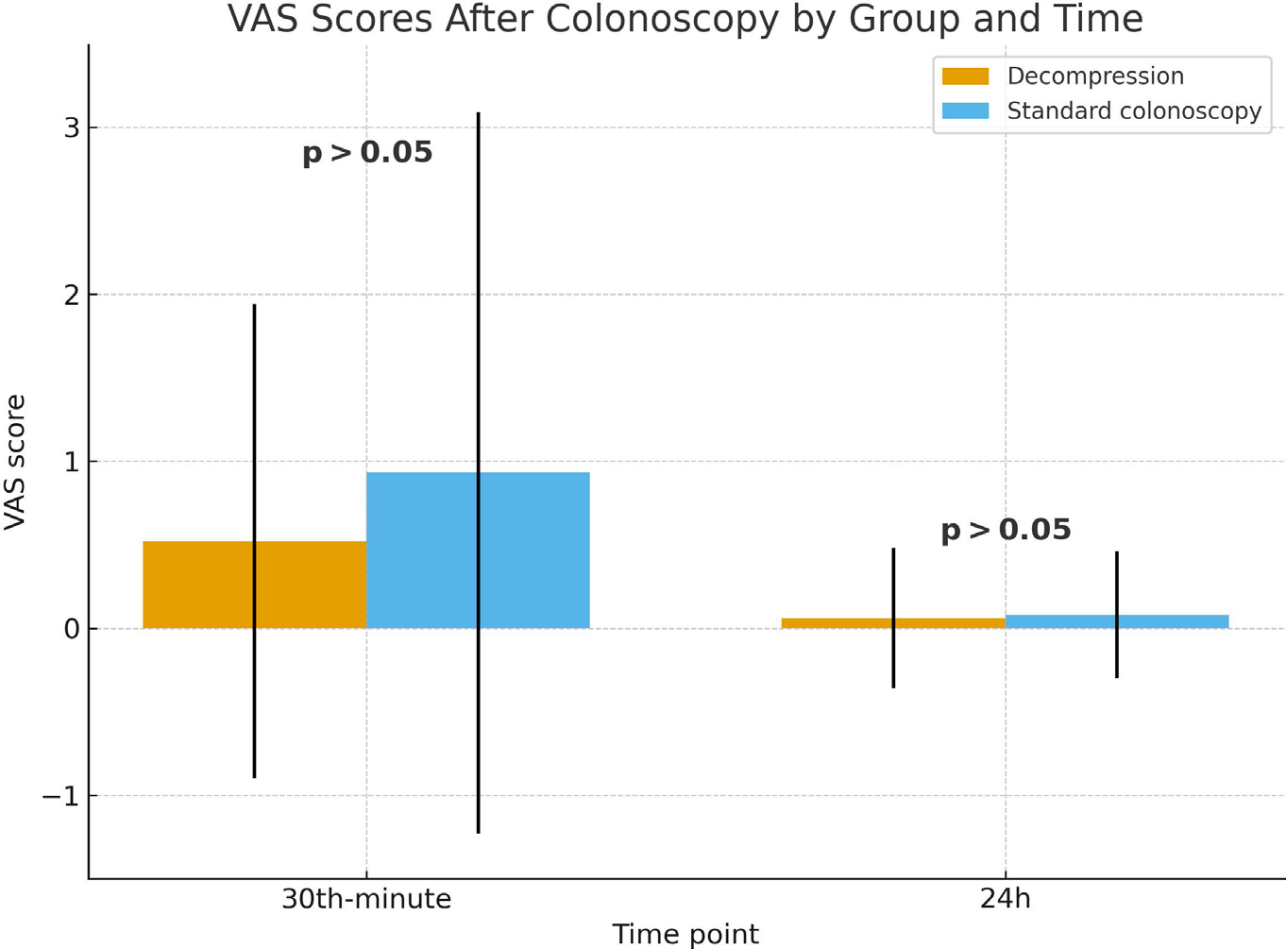
Comparison of 30-minute and 24-hour post-colonoscopy Visual Analog Scale scores between the decompression and standard colonoscopy groups.

At 24 hours post-colonoscopy, PCAP was reported in 2.6% of patients in the decompression group and 3.8% in the control group (*P* > .05). The mean VAS score at 24 hours was 0.06 ± 0.42 in the decompression group and 0.08 ± 0.38 in the control group (*P *= .779). These findings, including both early and 24-hour VAS scores, are illustrated in Figure 2.

In terms of pain severity, mild and moderate pain were observed in 66.7% and 33.3% of patients in the decompression group, respectively. In contrast, only mild pain was reported in the control group (100%). As shown in Table 2, the difference in pain severity between the groups was not statistically significant (*P* > .05).

When the demographic and clinical characteristics of patients were compared based on the presence or absence of PCAP, no statistically significant differences (*P* > .05) were found in terms of age, sex, BMI, smoking history, history of previous abdominal/gynecologic surgery, diagnosis of IBS, group assignment (decompression or control), administered propofol dose, quality of bowel preparation, heart rate, oxygen saturation, blood pressure, total procedure time, cecal intubation time, performance of biopsy and/or polypectomy, or need for abdominal compression (Table 3). However, a statistically significant difference (*P* = .008) was observed regarding comorbid disease history; patients with PCAP had a significantly lower frequency of comorbidities (Table 3).

### Analysis of Factors Associated with Pain

**Figure 3.**
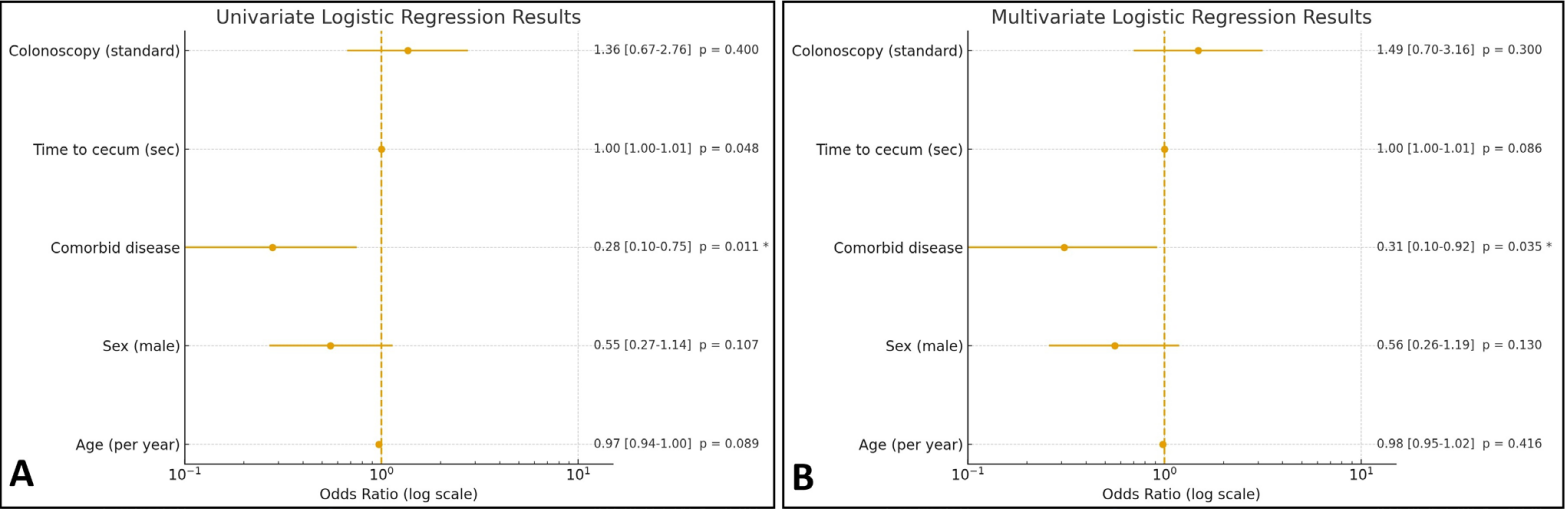
Logistic regression analyses of factors associated with post-colonoscopy abdominal pain. A: Univariate logistic regression model. B: Multivariate logistic regression model. Odds ratios are plotted on a logarithmic scale, with error bars indicating 95% CIs.

In this study, potential factors associated with PCAP were evaluated using both univariate and multivariate logistic regression analyses (Table 4). In the univariate analysis, the presence of comorbid disease (OR = 0.279, 95% CI: 0.104-0.751, *P *= .011) and cecal intubation time (OR = 1.003, 95% CI: 1.000-1.007, *P* = .048) were found to be statistically significant (Figure 3A). The presence of comorbidities appeared to act as a protective factor (OR < 1) against PCAP in this unadjusted analysis.

In the multivariate logistic regression model, comorbid disease remained significantly associated with a lower likelihood of PCAP (OR = 0.306, 95% CI: 0.102-0.918, *P* = .035). Cecal intubation time demonstrated only a borderline, non-significant trend toward increased pain (OR = 1.003, 95% CI: 1.000-1.007, *P* = .086) (Figure 3B). Age, sex, and type of colonoscopy procedure were not significantly associated with PCAP in either univariate or multivariate analyses (*P* > .05).

### Assessment of Complications

No major procedure-related complications, such as bleeding or perforation, were observed in either group during the early and late periods.

## Discussion

In this prospective, randomized controlled study, the effect of TCD on PCAP was evaluated. These results demonstrated that decompression of intraluminal air does not significantly reduce PCAP either in the early course or 24 hours after the procedure when compared with standard colonoscopy.

Adequate expansion of the colonic lumen is crucial for the identification of colonic pathologies during colonoscopy.^12^ However, residual gas as a result of air insufflation during the insertion and withdrawal of the colonoscope causes abdominal pain after the procedure.^16^ This pain is thought to arise from the stimulation of unmyelinated C fibers by residual colonic gas. Based on this mechanism, it has been hypothesized that TCD could reduce PCAP, leading to several studies investigating this concept.^7^^,^^11^^,^^12^ Two previous studies evaluating the effectiveness of TCD have yielded conflicting results. Lee et al^11^ demonstrated that decompression did not reduce PCAP. In contrast, Park et al^12^ reported that the frequency of abdominal pain was markedly lower in the decompression group (26.6% vs. 65.2%). These findings are consistent with those of Lee et al,^11^ suggesting that decompression does not have a clinically significant impact on PCAP. In another study evaluating the effect of decompression initiated from different colonic segments, PCAP was reported in 19% of patients who underwent decompression starting from the cecum, compared with 20.5% in those without decompression. Although this study demonstrated a significant difference in the severity of PCAP between the groups, which is consistent with the present findings, there was no meaningful difference in the incidence of abdominal pain.^7^

In the present study, the overall rate of PCAP among all participants was 16.9%. This value is considerably lower than the PCAP rate of up to 47.8% reported by Lee et al.^4^ in patients who received midazolam and alfentanil anesthesia. This difference may be primarily attributable to 2 factors: the procedural expertise of the single performing endoscopist and the combination anesthesia regimen employed in the current cohort, which included midazolam, remifentanil, and propofol. Since remifentanil was administered only as a 2.5 µg intravenous bolus and propofol lacks intrinsic analgesic properties, the impact of the anesthetic protocol on pain reduction may have been limited.^17^ The operator’s experience likely minimized scope looping and enabled gentle withdrawal maneuvers, both of which are known to reduce colonic wall tension and PCAP.^18^ Nevertheless, it should be noted that while using a single endoscopist ensured technical consistency, it may limit the generalizability of these findings.

This study demonstrated that the propofol dose administered to the decompression group was significantly higher than that administered to the control group. Although the time to colonoscope reinsertion in the cecum in the decompression group was approximately 50% shorter than the initial cecal intubation time (109.5 vs. 203 seconds), it was nevertheless associated with a higher dose requirement for propofol (196.54 ± 60.50 vs. 177.26 ± 38.98 mg). During colonoscopy, longer procedure times and higher propofol doses are associated with an increased risk of hypoxia.^19^ In this study, the lowest SpO_2 _levels observed in the decompression group were significantly lower than those in the control group, which was attributed to the substantially higher propofol doses administered (221.11 ± 79.12 vs. 194.42 ± 58.64 mg). Although statistically significant, the difference between the lowest SpO_2_ values has limited clinical relevance, as all values remained within a safe range and did not require intervention.

The use of CO_2_ insufflation in gastrointestinal endoscopy has become increasingly common. While meta-analyses have supported the routine use of CO_2_ insufflation over room air to reduce PCAP, the results of comparative studies have been conflicting.^6^^,^^20^^-^^25^ Several randomized controlled trials have evaluated this issue, with Mayr et al,^22^ Singh et al,^23^ and Chen et al^6^ demonstrating a significant reduction in PCAP with CO_2_ insufflation. In contrast, Szura et al^24^ and Gündüz et al^25^ reported no significant difference compared with room air. The continued reliance on room air is largely attributable to the lack of CO_2_ insufflators compatible with older endoscopy units and ongoing uncertainties regarding cost-effectiveness and cost–benefit.^20^ Based on these results, TCD did not prove effective in alleviating PCAP in settings where a CO_2_ insufflator was unavailable.

Previous studies have demonstrated that factors such as female sex, longer procedure duration, and low BMI are independent risk factors for the development of PCAP.^4^^,^^12^^,^^25^ In this study, multivariate analysis revealed that the presence of comorbidities was significantly associated with a lower likelihood of PCAP (OR = 0.31, 95% CI: 0.10-0.92, *P *= .035), while cecal intubation time showed only a borderline, non-significant trend toward increased pain (OR = 1.003, 95% CI: 1.000-1.007, *P* = .086). Comorbidities were present in 32.1% of the study population, the most common being hypertension (18.4%), followed by Type 2 diabetes (6.8%). Interestingly, the presence of comorbid disease was associated with lower odds of PCAP in both univariate and multivariate analyses. This relationship should be interpreted cautiously. Patients with comorbidities in the present cohort were significantly older, and age is known to influence pain perception and reporting, with younger individuals typically demonstrating greater pain sensitivity. Therefore, the observed negative association may reflect age-related differences in pain perception rather than a direct protective effect of comorbidity itself. This exploratory finding requires validation in future studies and should not be interpreted as a causal relationship.

In this study, although there was no significant difference in the polyp detection rate between the groups (33.3% vs. 30.8%), 9 participants (8%) in the decompression group had missed polyps. This rate is lower than the 16.8%-27.7% miss rates for polyps reported in second-look colonoscopy studies.^26^^,^^27^ The primary reason for this lower rate could be that, in the protocol, the endoscopist aimed to re-reach the cecum as quickly as possible to initiate air aspiration, without performing a detailed mucosal inspection during reinsertion.

All participants in the present study underwent ileal intubation, which allowed for complete visualization of the terminal ileum and ensured procedural consistency. This methodological approach distinguishes the present study from previous decompression studies investigating PCAP, in which ileal intubation was not specified.^7^^,^^11^^,^^12^ As a result, the current study better reflects real-life clinical practice and provides more reliable and comparable data. However, TCD primarily removes luminal air within the colon and does not eliminate gas that has already passed into the small intestine. Therefore, air retained in the small bowel could have contributed to the absence of a significant difference in PCAP between groups. Supporting this hypothesis, Lee et al^11^ reported that bloating scores were significantly reduced immediately post-colonoscopy but not at 24-48 hours, indicating that delayed bloating may be associated with air remaining in the small intestine rather than in the colon.

This study had several limitations. First, post-colonoscopy discomfort, characterized by bloating and gas, was not specifically assessed in the study population. Lee et al^11^ reported that decompression reduces postprocedural discomfort rather than pain. However, it could be said that abdominal discomfort has a less significant impact on patients’ willingness to undergo repeat colonoscopies compared to abdominal pain. Notably, the willingness to repeat colonoscopies was not evaluated in this study. Although all procedures were performed by a single experienced endoscopist to ensure technical consistency and eliminate inter-operator variability, this may limit the generalizability of the findings. Patients with cecal intubation times exceeding 10 minutes were excluded to enhance patient safety and comfort and to minimize confounding from procedure difficulty or excessive sedation dose. While this approach ensured standardized and comparable procedural conditions, it also represents a significant methodological limitation, as it may have prevented evaluation of the true relationship between technically difficult colonoscopies and PCAP. Although this study did not investigate colonoscopy quality indicators, recent evidence suggests that insertion-phase characteristics may influence overall procedural performance. Ishibashi et al^28^ reported that higher endoscopist satisfaction during scope insertion was associated with increased polyp and adenoma detection rates, highlighting the potential relevance of insertion-phase factors in colonoscopy practice.

In conclusion, these findings indicate that TCD has no beneficial effect in reducing PCAP. Although its impact on total procedure time was limited, the additional time required for decompression was associated with increased anesthetic drug use and a higher incidence of intraprocedural hypoxia.

## Figures and Tables

**Table 1.** Baseline Characteristics of the Groups

**Characteristics**	**Decompression Group ** **(n = 114)**	**Control Group ** **(n = 104)**	***P***
Age (years)	49.05 ± 11.78 ^a^	51.47 ± 10.82^ a^	.117 ^c^
Gender (Male/Female), n (%)	56 (49.1) / 58 (50.9)	53 (51) / 51 (49)	.786^ d^
BMI (kg/m^2^)	26.66 ± 4.46^ a^	26.49 ± 4.07 ^a^	.769 ^c^
Smoking, n (%)	39 (34.2)	32 (30.8)	.588 ^d^
Comorbid disease, n (%)	33 (28.9)	37 (35.6)	.295 ^d^
History of previous abdominal/gynecologic surgery, n (%)	24 (21.1)	24 (23.1)	.719 ^d^
History of IBS, n (%)	22 (19.3)	22 (21.2)	.733 ^d^
Sedation Propofol (mg) Midazolam (mg) Remifentanil (mcg)	196.54 ± 60.50 ^a^ 2 25	177.26 ± 38.98^ a^ 2 25	.005 *^,^^c^ N/A N/A
Bowel preparation, n (%) Fair Good Excellent	24 (21.1) 40 (35.1) 50 (43.9)	21 (20.2) 44 (42.3) 39 (37.5)	.524 ^d^
Pulse (beats/min) Pre-procedural Postprocedural	75.95 ± 11.44 ^a^ 71.76 ± 12.47 ^a^	74.12 ± 13.66 ^a^ 70.63 ± 10.94 ^a^	.283^ c^ .480^ c^
Oxygen saturation (%) Lowest Highest	94.89 ± 5.15 ^a^ 98.80 ± 2.23 ^a^	96.43 ± 2.83 ^a^ 99.01 ± 2.47 ^a^	.006 *^,^^c^ .509^ c^
Systolic blood pressure (mmHg) Pre-procedural Postprocedural	126.50 [115.50-143]^ b^ 112 [103-122] ^b^	125.50 [114.25-138] ^b^ 108 [97-119.75] ^b^	.423^ e^ .076^ e^
Diastolic blood pressure (mmHg) Pre-procedural Postprocedural	77 [67-84.25]^ b^ 68.50 [60.75-76]^ b^	75 [69-80] ^b^ 66 [58.25-73.75]^ b^	.298^ e^ .216^ e^
Time to reach cecum (s)	203 [141-277.50] ^b^	222.50 [153-318.25] ^b^	.089^ e^
Total procedure time (s)	737 [580.75-891.75] ^b^	738 [612.25-931.75]^ b^	.327^ e^
Reinsertion time (s)	109.50 [80.75-167] ^b^		
Biopsy performed, n (%)	13 (11.4)	8 (7.7)	.354^ d^
Endoscopic resection performed, n (%)	38 (33.3)	32 (30.8)	.685^ d^
Abdominal compression, n (%)	10 (8.8)	20 (19.2)	.025 *^,^^d^
Position change, n (%)	0 (0)	0 (0)	N/A

BMI, body mass index; IBS, irritable bowel syndrome; N/A, not applicable.

^a^Mean ± SD.

^b^Median (interquartile difference).

^c^Independent *t*-test.

^d^Chi-square test.

^e^Mann–Whitney *U*-test.

*Values given in bold are statistically significant (*P* < .05).

**Table 2.** Frequency and Severity of Post-Colonoscopy Abdominal Pain According to Groups

**Characteristics**	**Decompression Group ** **(n = 114)**	**Control Group ** **(n = 104)**	*P*
Early post-colonoscopy pain, n (%)	17 (14.9)	20 (19.2)	.396 ^b^
VAS score	0.52 ± 1.42^ a^	0.93 ± 2.16^ a^	.100^c^
Pain severity, n (%) Mild Moderate Severe	10 (58.8) 5 (29.4) 2 (11.8)	6 (30) 9 (45) 5 (25)	.201 ^b^
Pain 24h after colonoscopy, n (%)	3 (2.6)	4 (3.8)	.712 ^d^
VAS score	0.06 ± 0.42^ a^	0.08 ± 0.38 ^a^	.779 ^c^
Pain severity, n (%) Mild Moderate Severe	2 (66.7) 1 (33.3) 0 (0)	4 (100) 0 (0) 0 (0)	.429 ^d^

VAS, Visual Analog Scale.

^a^Mean ± SD.

^b^Chi-square test.

^c^Independent *t*-test.

^d^Fisher’s Exact test.

**Table 3.** Comparison of Demographic and Clinical Characteristics of Patients According to Abdominal Pain Status After Colonoscopy

**Characteristics**	**Patients with Pain ** **(n = 37) **	**Patients Without Pain ** **(n = 181)**	*P*
Age (years)	47.30 ± 10.34 ^a^	50.80 ± 11.50 ^a^	.088 ^c^
Gender (Male/Female), n (%)	23 (62.2) / 14 (37.8)	86 (47.5) / 95 (52.5)	.104^ d^
BMI (kg/m^2^)	25.98 ± 4.37^ a^	26.70 ± 4.25 ^a^	.350 ^c^
Smoking, n (%)	13 (35.1)	58 (32)	.715 ^d^
Comorbid disease, n (%)	5 (13.5)	65 (35.9)	.008 *^,^^d^
History of previous abdominal/gynecologic surgery, n (%)	9 (24.3)	39 (21.5)	.710 ^d^
History of irritable bowel syndrome, n (%)	7 (18.9)	37 (20.4)	.833 ^d^
Patients group, n (%) Decompression Control	17 (45.9) 20 (54.1)	97 (53.6) 84 (46.6)	.396 ^d^
Sedation Propofol (mg) Midazolam (mg) Remifentanil (mcg)	180 [160-205] ^b^ 2 25	180 [150-200] ^b^ 2 25	.405^ e^ N/A N/A
Bowel preparation, n (%) Fair Good Excellent	7 (18.9) 13 (35.1) 17 (45.9)	38 (21) 71 (39.2) 72 (39.8)	.785 ^d^
Pulse (beats/min) Pre-procedural Postprocedural	76.45 ± 15.31 ^a^ 72.32 ± 14.19 ^a^	74.79 ± 11.94 ^a^ 71.00 ± 11.22 ^a^	.283^ c^ .480^ c^
Oxygen saturation (%) Lowest Highest	95.16 ± 4.20^a^ 98.57 ± 3.93^a^	95.72 ± 4.29^a^ 98.97 ± 1.88^a^	.464^c^ .550^c^
Systolic blood pressure (mmHg) Pre-procedural Postprocedural	126 [114.50-138.50]^ b^ 113 [100.50-124.50] ^b^	126 [114.50-142] ^b^ 110 [101-120.50] ^b^	.618^ e^ .497^ e^
Diastolic blood pressure (mmHg) Pre-procedural Postprocedural	74 [69.50-79.50]^ b^ 68 [63-78]^ b^	76 [67-83] ^b^ 68 [60-74]^ b^	.433^ e^ .230^ e^
Time to reach cecum (s)	268 [157.50-333] ^b^	203 [142-286.50] ^b^	.056^ e^
Total procedure time (s)	751 [616.50-920] ^b^	735 [599.50-904]^ b^	.643^ e^
Reinsertion time (s)	99 [81.50-166.50] ^b^	113 [79-167] ^b^	.892^ e^
Biopsy performed, n (%)	3 (8.1)	18 (9.9)	1.000^ f^
Endoscopic resection performed, n (%)	11 (29.7)	59 (32.6)	.734 ^d^
Abdominal compression, n (%)	7 (18.9)	23 (12.7)	.318 ^d^
Position change, n (%)	0 (0)	0 (0)	N/A

BMI, body mass index; N/A, not applicable.

^a^Mean ± SD.

^b^Median [Interquartile Difference].

^c^Independent *t*-test.

^d^Chi-square test.

^e^Mann–Whitney *U*-test.

^f^Fisher’s Exact test.

*Values given in bold are statistically significant (*P* < .05).

**Table 4.** Univariate and Multivariate Logistic Regression Analysis of Factors Associated with Post-Colonoscopy Abdominal Pain

**Factors**	**Univariate OR (95% CI)**	***P***	**Multivariate OR (95% CI)**	*P*
Age (years)	0.972 (0.941-1.001)	.089	0.984 (0.946-1.023)	.416
Gender (Male)	0.551 (0.267-1.138)	.107	0.556 (0.261-1.188)	.130
Comorbid disease	0.279 (0.104-0.751)	.011 *	0.306 (0.102-0.918)	.035 *
Cecum reaching time (s)	1.003 (1.000-1.007)	.048 *	1.003 (1.000-1.007)	.086
Colonoscopy type (standard)	1.359 (0.668-2.762)	.397	1.489 (0.702-3.160)	.300

OR, odds ratio.

*SValues given in bold are statistically significant (*P* < .05).

## Data Availability

The data that support the findings of this study are available on request from the corresponding author.
